# Editorial: Evolutionary dynamics, functional variation and application of plant organellar genomes

**DOI:** 10.3389/fpls.2026.1811316

**Published:** 2026-03-09

**Authors:** Zhiqiang Wu, Luke R. Tembrock

**Affiliations:** 1 Guangdong Laboratory of Lingnan Modern Agriculture, Key Laboratory of Synthetic Biology, Ministry of Agriculture and Rural Affairs, Agricultural Genomics Institute at Shenzhen, Chinese Academy of Agricultural Sciences, Shenzhen, China; 2 Department of Agricultural Biology, Colorado State University, Fort Collins, CO, United States

**Keywords:** chloroplast genome, mitochondrial genome, genome assembling, comparative genomics, phylogeny construction

## 1 Introduction

Organellar genomes of plants are semi-autonomous genetic systems housed within chloroplasts and mitochondria and provide key data in deciphering the plant tree of life, adaptive evolution, and environmental interactions (Smith and Keeling, 2015; Wang et al., 2024a). The chloroplast genome, with its relatively conserved structure and uniparental inheritance, has long served as a cornerstone for phylogenetic and biogeographic studies (Li et al., 2019). In contrast, the mitochondrial genome is renowned for its astonishing structural dynamism, including frequent rearrangements, abundant repetitive sequences, and active intracellular DNA transfers (Wang et al., 2024b). These characteristics present significant challenges for assembly and analysis while simultaneously offering a unique window into genome evolution and functional innovation. For many years, the study of these organelles, particularly mitochondria, was constrained by sequencing technologies. However, the convergence of high-throughput long-read sequencing and advanced bioinformatic algorithms has revolutionized the field, enabling the precise reconstruction of complex organellar architectures (Bi et al., 2024; He et al., 2023; Xian et al., 2025; Tang et al., 2025).

In August 2024, Dr. Zhiqiang Wu (Agricultural Genomics Institute at Shenzhen, Chinese Academy of Agricultural Sciences, China), Dr. Andan Zhu (Kunming Institute of Botany, Chinese Academy of Sciences, China), Dr. Luke R Tembrock (Colorado State University, USA) and Dr. Changwei Bi (Nanjing Forestry University, China) organized an issue entitled “Evolutionary Dynamics, Functional Variation and Application of Plant Organellar Genome” in Frontiers in Plant Science under the Plant Bioinformatics section to highlight recent advances in organellar genome research. For one-year submissions were expected for this Research Topic which culminated in 31 original research studies that were contributed by an extensive and active international collaborative network. The contributions of research involved 93 independent research institutions from 58 cities across eight countries (China, Singapore, Germany, Switzerland, Australia, United States, Republic of Korea, Japan). Institutions in China were the primary contributors, with contributions coming mainly from research units in Beijing, Kunming, Shanghai, Nanjing, Wuhan, Guiyang, and Hangzhou. Collaborations with institutions in Germany (Technical University of Munich), Switzerland (University of Fribourg), the United States (Colorado State University), Australia (Murdoch University), Singapore (Nanyang Technological University), Japan (National Museum of Nature and Science), and the Republic of Korea (Chungnam National University) reflect the international perspective and high level of cooperation in this field. This cross-regional, inter-institutional collaborative model has been vital in driving the rapid, groundbreaking progress in plant organellar genomics.

This Research Topic brings together 31 original research articles that collectively mark a new stage of development in plant organellar genomics. Spanning a broad spectrum of plant taxa and ecological contexts—from foundational genome mapping to in-depth comparative and evolutionary analyses—these studies provide novel insights into genome structure, dynamic evolution, selective pressures, and phylogenetic relationships. By expanding available genomic resources and analytical frameworks, this Research Topic not only deepens our fundamental understanding of organellar biology but also furnishes critical tools for species conservation, molecular breeding, and biodiversity research. The following sections will synthesize the key findings, outline the extensive international collaborative network behind them, and discuss future research directions.

## 2 Organellar genome features and structural diversity

A core contribution of this Research Topic is the significant expansion of the plant organellar genome database, particularly for many previously unsequenced taxa. The main findings include: (1) Structural Expansion and Innovation in Chloroplast Genomes: this research reveals that chloroplast genomes are not static. For instance, in the legume *Calliandra haematocephala*, the expansion of the inverted repeat (IR) region into the large single copy (LSC) region by approximately 14 kb, coupled with a high abundance of clustered dispersed repeats, has resulted in the largest plastome (200,623 bp) reported to date in Mimoseae and, more broadly, in Leguminosae. Similarly, in *Scaevola* species (Goodeniaceae), plastome length increased by ~30 kb through IR expansion and LSC fragment duplication. These findings challenge the traditional view of chloroplast genomes as highly conserved and suggest that repetitive sequences play an active role in driving structural variation and size changes. (2) Complex Conformations of Mitochondrial Genomes: multiple studies report the first mitochondrial genomes for various species, highlighting their astounding structural diversity. For example, the mitogenome of *Populus kangdingensis* consists of three independent circular molecules, whereas that of *P. ciliata* exhibits a branched structure comprising both circular and linear molecules. The mitogenomes of *Magnolia kwangsiensis*, *Camellia oleifera*, and *C. meiocarpa* also display multi-branched or complex multi-molecular configurations. The mitochondrial genome of *Iris domestica* is comprised of four contigs that, mediated by large repeat sequences, can form three circular chromosomes, suggesting a possible multi-chromosomal structure. Collectively, these studies indicate that plant mitochondrial genomes are far from simple “master circles”; in fact complex conformations may be the norm rather than the exception. (3) Repetitive Sequences and Inter-Organellar DNA Transfer: repetitive sequences (dispersed, tandem, and simple sequence repeats) are ubiquitously identified as major drivers of recombination, expansion, and structural variation in organellar genomes. For instance, the mitogenome of *Distylium racemosum* contains the highest repeat content reported to date among Saxifragales mitogenomes. Concurrently, DNA transfer events from the chloroplast to the mitochondrion are detected in almost all studies. These transferred fragments, known as mitochondrial plastid DNAs (MTPTs), constitute a variable percentage of the mitogenome length, ranging from 2.81% in *Lycopodium japonicum* to over 13% in *Rubus chingii* var. *suavissimus*. These homologous sequences not only enrich the mitochondrial genome’s composition but may also influence evolution by providing new regulatory elements or functional gene fragments.

## 3 Comparative and evolutionary genomics

Building upon genome maps, in-depth comparative analyses reveal evolutionary forces and signals of adaptation: (1) Selective Pressures and Adaptive Evolution: analyses of the non-synonymous to synonymous substitution rate ratios (Ka/Ks) show that most protein-coding genes are under purifying selection, maintaining their core functions. More strikingly, signals of positive selection are detected in specific environmental contexts. For example, in high-altitude species, genes such as *matK* and *ndhB* in Rosaceae, *atp4*, *ccmB*, and *mttB* in *Populus*, and *atp6*, *ccmB*, *nad4L*, and *nad7* in *Hippophae tibetana* show signs of positive selection, suggesting their crucial roles in adapting to high altitude environments. Similarly, in the mangrove associate *Dolichandrone* sp*athacea*, 12 chloroplast genes related to photosystem-associated proteins bear signatures of positive selection, potentially linked to adaptation to the intertidal zone. These findings help connect genomic variation to specific ecological adaptations. (2) Ubiquity and Tissue-Specificity of RNA Editing: RNA editing (predominantly C-to-U conversions) is a key post-transcriptional regulatory mechanism in plant organellar gene expression. Studies in this Research Topic systematically predict a vast number of RNA editing sites, e.g., 600 sites in the *Citrus medica* mitogenome and 408 in *Leonurus japonicus*. Notably, research on *Lonicera macranthoides* reveals significant differences in RNA editing sites across tissues (leaves, flowers, stems), indicating tissue-specific regulation that may fine-tune developmental or physiological processes. (3) Codon Usage Bias: Most studies analyzed the Relative Synonymous Codon Usage (RSCU), commonly finding an A/T bias at the third codon position. This reflects the interplay of translation efficiency by nucleotide composition bias and potential natural selection.

## 4 Phylogenetics and taxonomic applications

Organellar genomes provide powerful datasets for resolving long-standing phylogenetic controversies and clarifying taxonomic relationships: (1) Clarifying Higher-Order Phylogenetic Relationships: phylogenetic analyses based on complete chloroplast genomes provide robust evolutionary frameworks for several groups. For example, studies strongly support the division of Zingiberaceae into two primary subfamilies (Alpinioideae and Zingiberoideae) and clarify key relationships among genera like *Globba*, *Curcuma*, and *Hedychium*. Within Rosaceae, phylogenetic reconstruction using organellar genomes unravels the complex reticulate evolutionary history of *Sorbus* sensu lato, demonstrating the non-monophyletic status of the core genus *Sorbus* due to the nested placement of hybrid-origin genera *Hedlundia* and *Scandosorbus*. (2) Taxonomic Insights and Revision: genomic data provide direct evidence for specific taxonomic revisions. Chloroplast phylogenomics indicates that species of the medicinal genus *Agapetes* are nested within *Vaccinium*, a conclusion further supported by nuclear ITS sequences and codon usage patterns, thereby offering key molecular evidence for merging these two important medicinal genera. Similarly, studies identify potential DNA barcode regions for distinguishing species within *Weigela* and *Dolichandrone*. (3) Organellar Phylogenetic Incongruence: several studies note topological inconsistencies between phylogenies constructed from chloroplast and mitochondrial genomes (e.g., in *Prunus*, *Camellia*). Such incongruence, potentially arising from incomplete lineage sorting, hybridization events, horizontal gene transfer, or differing evolutionary rates, is itself an important insight into complex evolutionary histories.

## 5 Future organellar genome research perspectives

Based on the advances and remaining challenges showcased in this Research Topic, future research in plant organellar genomics should expand in the following directions: (1) From Correlation to Causation: The Imperative for Functional Validation: current studies, using comparative genomics, have identified a plethora of putative adaptive genetic variants, including positively selected genes, specific RNA editing sites, and structural variations. The crucial next step is to employ moleular tools—such as gene editing (e.g., CRISPR; TALEN), transgenic complementation, and proteomics—in model or closely related systems to validate the physiological impact of these variants (Krämer et al., 2024). Establishing causal links between specific genotypes and phenotypic traits (e.g., stress tolerance, metabolism, and development) within an environmental context is essential to move beyond associative interpretations. (2) Integrating Multi-Omics and Dynamic Regulation Studies: future research must transcend static descriptions of genome structure. Integrating transcriptomic, proteomic, metabolomic, and epigenomic (e.g., organellar DNA methylation) data will be vital to unravel the dynamic regulatory networks governing organellar gene expression across tissues, developmental stages, and environmental stresses (Xiang et al., 2026). This integrated approach is key to understanding the complex signaling dialogue and co-evolution between the nucleus and organelles.

## 6 Technological innovation and standardization

Even though thousands of finished organellar genomes are available at present, additional innovations and advancements are needed to overcome various technical challenges. First, Overcoming Assembly Hurdles. For ultra-large, highly repetitive, and multi-conformational mitochondrial genomes, further optimization of sequencing strategies (e.g., ultra-long reads, Hi-C scaffolding) and algorithms is needed to achieve complete, end-to-end, and error-free assemblies. Second, Single-Cell and Spatial Organellar Genomics. Applying single-cell sequencing technologies holds promise for revealing heterogeneity in organellar genomes among cells within the same tissue. This is crucial for understanding cytoplasmic inheritance, somatic mutations, and other physiological phenomena. Third. Data Standardization and Sharing. Establishing unified annotation standards, quality assessment metrics, and open-access databases will significantly facilitate cross-study and cross-species comparative analyses, accelerating discovery. Fourth, Filling Taxonomic Gaps. Future work should extend to more basal plant lineages (e.g., bryophytes, algae), parasitic plants, and ecologically keystone species that are currently underrepresented. This will enable the reconstruction of a more complete panorama of plant organellar genome evolution. Last, Deepening Ecology-Evolution Integration. Integrating organellar genomic variation data with species distribution models, population demographic history, and paleoclimatic data will allow for quantitative assessment of how geological and historical events (e.g., Qinghai-Tibet Plateau uplift, glacial oscillations) have shaped the evolutionary trajectories and adaptive diversification of plant organellar genomes.

## 7 Conclusion

The 31 studies in this Research Topic are like 31 pieces of a puzzle, collectively forming a vibrant picture of the current state of plant organellar genomics. They have not only substantially enriched the organellar genome repository spanning from basal vascular plants to higher angiosperms but have also deepened our understanding across multiple dimensions. From recognizing the extreme complexity of mitochondrial genome structure to revealing the central driving force of repeats and inter-organellar sequence transfer in genome evolution. From uncovering molecular imprints of adaptation to extreme environments like high altitudes and coastal environments at the genomic level, to utilizing whole-genome data to resolve intricate phylogenetic relationships. These achievements are inseparable from the close collaboration of global researchers and continuous technological advancement.

Looking ahead, plant organellar genomics stands at a turning point, transitioning from a “descriptive science” to a “mechanistic science.” Future research will undoubtedly place greater emphasis on functional validation, multi-omics integration, technological innovation, and links to macro-evolutionary ecology. We anticipate that the continued exploration of the genomic mysteries within these semi-autonomous cellular “power stations” and “photosynthetic factories” will provide a deeper theoretical foundation and a more powerful toolkit for understanding the origins of plant diversity, addressing biodiversity conservation under global climate change, and promoting sustainable agricultural development. This Research Topic represents a solid and significant step toward this new era.
